# The Process–Property–Performance Relationship of Medicated Nanoparticles Prepared by Modified Coaxial Electrospraying

**DOI:** 10.3390/pharmaceutics11050226

**Published:** 2019-05-10

**Authors:** Weidong Huang, Yuan Hou, Xinyi Lu, Ziyun Gong, Yaoyao Yang, Xiao-Ju Lu, Xian-Li Liu, Deng-Guang Yu

**Affiliations:** 1 School of Chemistry and Chemical Engineering, Hubei Polytechnic University, Huangshi 435003, China; neweydong@hbpu.edu.cn; 2 Hubei Key Laboratory of Mine Environmental Pollution Control and Remediation, School of Environmental Science and Engineering, Hubei Polytechnic University, Huangshi 435003, China; 3 School of Materials Science and Engineering, University of Shanghai for Science and Technology, Shanghai 200093, China; 1626410104@st.usst.edu.cn (Y.H.); 1626418101@st.usst.edu.cn (X.L.); 1626410115@st.usst.edu.cn (Z.G.); yyyang@usst.edu.cn (Y.Y.)

**Keywords:** coaxial electrospraying, polymeric nanoparticles, spreading angle, process-property-performance relationship

## Abstract

In pharmaceutical nanotechnology, the intentional manipulation of working processes to fabricate nanoproducts with suitable properties for achieving the desired functional performances is highly sought after. The following paper aims to detail how a modified coaxial electrospraying has been developed to create ibuprofen-loaded hydroxypropyl methylcellulose nanoparticles for improving the drug dissolution rate. During the working processes, a key parameter, i.e., the spreading angle of atomization region (*θ*, °), could provide a linkage among the working process, the property of generated nanoparticles and their functional performance. Compared with the applied voltage (*V*, kV; *D* = 2713 − 82*V* with *R*_θV_^2^ = 0.9623), *θ* could provide a better correlation with the diameter of resultant nanoparticles (*D*, nm; *D* = 1096 − 5*θ* with *R*_Dθ_^2^ = 0.9905), suggesting a usefulness of accurately predicting the nanoparticle diameter. The drug released from the electrosprayed nanoparticles involved both erosion and diffusion mechanisms. A univariate quadratic equation between the time of releasing 95% of the loaded drug (*t*, min) and *D* (*t* = 38.7 + 0.097*D* − 4.838 × 10^5^*D*^2^ with a *R*^2^ value of 0.9976) suggests that the nanoparticle diameter has a profound influence on the drug release performance. The clear process-property-performance relationship should be useful for optimizing the electrospraying process, and in turn for achieving the desired medicated nanoparticles.

## 1. Introduction

Today, nanomaterials play one of the most important roles in the research and development of modern pharmaceutics [1,2,3]. New material processing procedures [4,5,6,7,8], combined with different kinds of raw materials [9,10,11,12,13] and novel innovative strategies for constructing functional products [14,15,16,17,18], are frequently introduced into this application field for providing efficacious drug delivery and enhancing the therapeutic effects of active pharmaceutical ingredients (APIs). Among them, electrohydrodynamic atomization (EHDA) is a popular technique for creating nanoproducts, which mainly includes electrospraying and electrospinning. These new methods explore electrical energy to atomize the working fluid for producing solid products at micro or nano scale [19,20,21,22,23].

The past two decades have witnessed the rapid progress of electrosprayed nanoparticles being utilized as functional products in a wide variety of fields [24,25,26,27,28,29,30]. In pharmaceutics, further developments of medicated electrosprayed nanoparticles include creating complex nanostructures (just as its counterpart electrospinning [31,32,33,34,35,36]), production on large scales, potential clinical applications, and commercial products [37,38]. However, the electro–hydro–dynamic working process is still far from being understood, due to the overlap of several disciplines such as fluid mechanics, electric dynamics, and polymer rheology during the extremely fast drying processes of electrospraying [39,40]. Even a purposeful and conscious manipulation of the electrosprayed nanoparticle’s diameter is very hard to realize.

**Figure 1:**
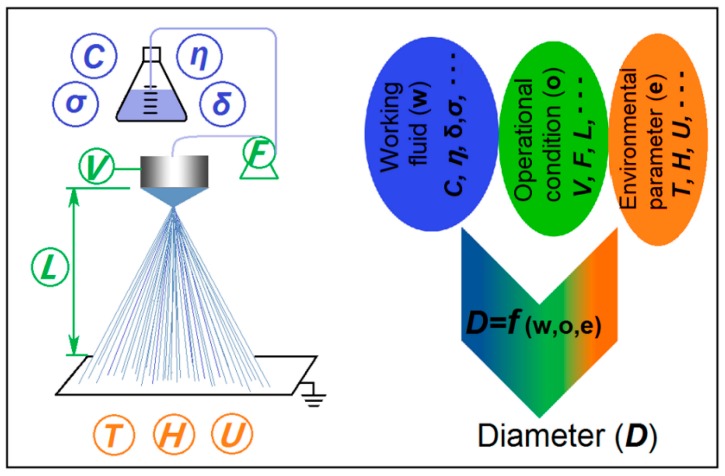
A diagram of the single-fluid electrospraying process and the experimental parameters exerting influence on the diameter of resultant nanoparticle.

Shown in Figure 1 is a diagram about the single-fluid electrospraying process and the possible experimental parameters that can exert significant influences on the diameters of resultant nanoparticles. An electrospraying apparatus brings together the working fluid and electrostatic energy at the convergent point, i.e., the nozzle of spraying head. Between the two electrodes consisting of spraying head and collector, the working fluids are atomized and solidified into particles within several decades of microseconds. Based on this, all the experimental parameters can be divided into three categories which are concluded in Figure 1. Correspondingly, the resultant nanoparticles’ diameter (*D*) can be a function of working fluid’s property (*w*), operation conditions (*o*), and environmental parameters (*e*), i.e., *D* = *f* (w,o,e).

During the past several decades, numerous publications have investigated the influence of particular parameters on electrosprayed products. These articles disclosed the process-property relationship for intentionally manipulating the particles’ diameters, morphology, and surface smoothness [41,42,43]. However, there are too many parameters that can exert significant influence on the final products during the electrospraying processes [44,45]. For example, the properties of working fluid include polymer concentration (*C*), viscosity (*η*), surface tension (*δ*), and also conductivity (*σ*). The operational parameters include the applied voltage (*V*), the fluid flow rate (*F*), and also the particle collected distance (*L*). The environmental conditions include temperature (*T*), humidity (*H*), possible vacuum (*U*), and sometime with hot air blowing.

Thus, it is difficult to manipulate the diameter of final nanoparticles accurately through particular experimental parameters. In contrast, the parameters of the electrospraying itself seem to be neglected. Compared with the experimental parameters that can be controlled directly by researchers, very few publications have reported uncontrollable parameters in relation to working processes, such as the size and angle of a Taylor cone, the length of straight fluid jets, and the spreading angle of the atomization region.

Based on the above-mentioned knowledge, here for the first time, we have investigated the influence of spreading an angle of the atomization region on the diameter of resultant nanoparticles. Meanwhile, the influence of applied voltage on the spreading angle and nanoparticles’ diameters, and the size of medicated nanoparticle on the drug fast release performance were also studied. Thus, an example about how to disclose the process-property-performance relationship of medicated nanoparticles prepared by modified coaxial electrospraying is showed. In the experiments, ibuprofen (IBU) and hydroxypropyl methylcellulose (HPMC) were selected as the model drug and polymer matrix, respectively. IBU, a typical nonsteroidal anti-inflammatory drug, is broadly exploited to treat pain, fever and inflammation. However, its poor water solubility always limits its fast action for achieving a desired therapeutic effect [46,47,48]. HPMC is commonly utilized as an excipient in oral tablet, eye drops, and capsule formations. It can be used both as a delaying agent for controlled release, as well as an enhancer to improve the soluble rate of a soluble drug [49,50].

## 2. Materials and Methods

### 2.1. Materials

IBU was provided by Wuhan Anruike Biological Pharmaceutical Co., Ltd. (Wuhan, China). HPMC powders (2910 5cps, *M*_n_ = 428,000 g/mol, methoxy content = 28.0–30.0%, hydroxypropoxy content = 7.5–12%) were obtained from Shandong Fine Chemical Co., Ltd. (Jinan, China). Ethanol and dichloromethane (DCM) were purchased from Shanghai Lingfeng Chemical Testing Co. Ltd. (Shanghai, China). All other chemicals are analytical reagents, and water was distilled twice before use.

### 2.2. Modified Coaxial Electrospraying

A solidifiable solution consisting of 7% (*w*/*v*) HPMC and 3% (*w*/*v*) IBU in a mixture of ethanol and DCM (1:1, *v*:*v*) was prepared and utilized as the core fluid. Pure solvent ethanol was used as the shell fluid. Four nanoparticles referred to as P1, P2, P3, and P4 were prepared at an applied voltage of 16, 17, 18, and 19 kV, respectively. For all preparations, the particle-collected distance was fixed at 15 cm. The shell and core fluid flow rates were 0.2 and 0.8 mL/h, respectively. The electrospraying processes were recorded using a digital camera (PowerShot A640, Tokyo, Japan).

### 2.3. Morphology of the Prepared Nanoparticles

The surface morphological characterization of the prepared nanoparticles was observed under scanning electron microscopy (SEM; Quanta FEG450, FEI Corporation, Hillsboro, OR, USA) at 20 kV of accelerated voltage. Before the observation, the samples were sputter-coated with gold under vacuum. The images were analyzed by ImageJ software with the measuring of over 100 different nanoparticles and their diameters presented as mean ± S.D.

### 2.4. Drug Fast Release Performance

An amount of 20 mg medicated nanoparticles was placed in 50 mL of phosphate buffer solution (PBS) with a pH value of 7.0. The buffer solutions including samples were incubated in a shaking bath at 37 ± 0.1 °C and an agitation speed of 50 rpm. Each type of sample was repeated 6 times. At predetermined time intervals, 1 mL of sample solutions were withdrawn and replaced with 1 mL fresh medium. The amount of IBU released from the nanofibers was measured using a UV-Vis Spectrophotometer (UV-2102PC, Unico Instrument Co. Ltd., Shanghai, China) by measuring the absorbance at 264 nm. The calibration curve was obtained, and all concentrations were evaluated in percentage as mean ± S.D. using Equation (1).

(1) $$Accumulative\;release\;(\%)=\frac{Amount\;of\;drug\;release}{Amount\;of\;initial\;drug}\times 100$$

## 3. Results and Discussion

### 3.1. Preparations of the Medicated Particles Using the Modified Coaxial Electrospraying

**Figure 2:**
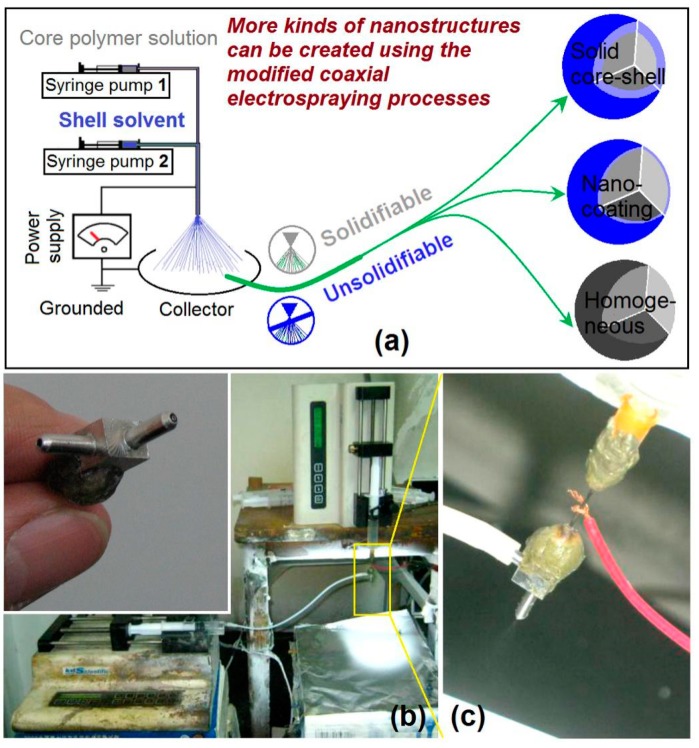
The modified coaxial electrospraying: (**a**) A diagram about the process, by which many kinds of nanostructures can be created through the unsolidifiable shell fluids; (**b**) implementation of the modified coaxial electrospraying, the upper-left inset shows the home-made concentric spraying head; (**c**) the connection of power supply and working fluid with the spraying head.

A diagram about the modified coaxial electrospraying process is shown in Figure 2a. Right from the first publication about coaxial electrospraying [25], all the shell fluids must be solidifiable to ensure the creation of core-shell nano-/micro-structures. However, this limitation was broken by Yu and his co-worker. They reported a modified coaxial process, in which fluids without solidifiable properties using a single-fluid electrospraying process can be introduced into the coaxial process and was explored as the shell working fluids [43,44,45]. This new process greatly expanded the capability of electrospraying as it was deemed capable of generating additional kinds of nanostructures, including core-shell solid structure, nanocoating and homogeneous particles with a high quality (Figure 2a).

The organization of electrospraying systems and the implementation of an electrospraying process are exhibited in Figure 2b. A home-made concentric spraying head was exploited to implement the modified coaxial electrospraying (the upper-left inset of Figure 2b), which can also be utilized to conduct coaxial electrospinning [49]. The inner metal capillary has an inner and outer diameter of 0.3 mm and 0.6 mm, respectively. The orifice of the outer capillary has a diameter of 1.2 mm. Two syringe pumps (KDS100 and KDS 200, Cole-Parmer^®^, Vernon Hills, IL, USA) were employed to drive the core and shell liquids to the spraying head (Figure 2b). A copper line was directly attached on the concentric spraying head to convey the electrostatic energy to the working fluids (Figure 2c).

**Figure 3:**
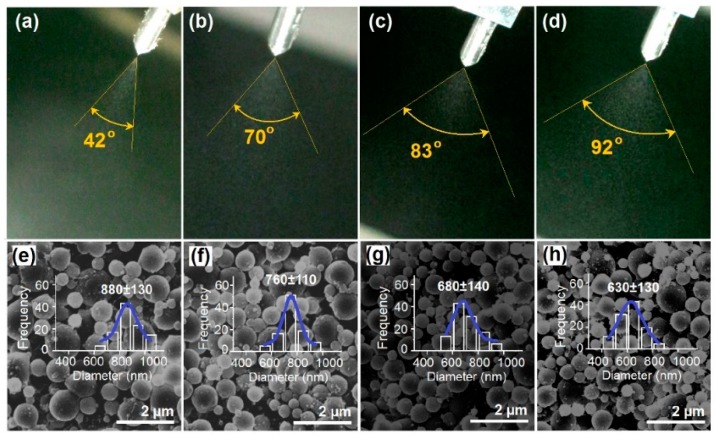
(**a**–**d**) The typical changes of spreading angles with the elevation of applied voltages (kV) from 16, to 17, 18, and 19, respectively (*n* = 3); (**e**–**h**) SEM images of the resultant nanoparticles and their diameter distributions (**e**) P1; (**f**) P2; (**g**) P3; (**h**) P4.

During the EHDA process, the applied voltage is one of the most important parameters that have significant influence on the final products. As for the working fluids systems, there are often suitable working ranges for almost all the experimental parameters. In the present systems, when the applied voltages were changed from 16 kV to 19 kV, all the coaxial electrospraying processes were run continuously, smoothly and robustly. With an enlarged shooting, the images of Taylor cones are shown in Figure 3a–d. The estimated spreading angles of three measurements are (42 ± 3)°, (70 ± 6)°, (83 ± 5)°, and (92 ± 4)°, for an applied voltage of 16, 17, 18 and 19 kV, respectively. As the voltage elevated, the spreading angles increased correspondingly. For reference, to achieve the clearest images these photos were taken under a certain tilted angle, however the camera lens should always keep the same height as the nozzle of a spraying head.

The SEM images of the prepared nanoparticles and their diameter distributions are shown in Figure 3e–h. Nanoparticles P1, P2, P3, and P4 have an estimated diameter of 880 ± 130, 760 ± 110, 680 ± 140, and 630 ± 130 nm, respectively. All the nanoparticles have a round surface with some satellites. As the applied voltages increased, the diameters decreased correspondingly.

### 3.2. The Process–Property Relationship and the Related Mechanism

**Figure 4:**
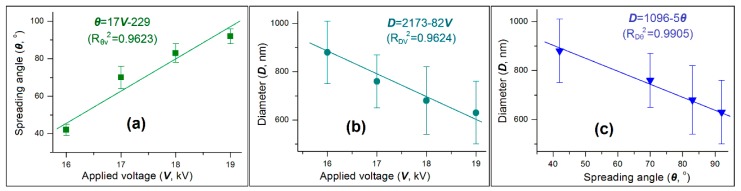
The applied voltage-spreading angle-nanoparticle diameter relationships: (**a**) The influence of applied voltage on the spreading angle; (**b**) the influence of applied voltage on the nanoparticles’ diameter; (**c**) the accurate relationship between the spreading angle and the nanoparticles’ diameter.

Shown in Figure 4a is the influence of applied voltage on the spreading angle. The trend is clear that the spreading angle increased as the applied voltage elevated. A linear regression suggests these two parameters have a relationship of *θ* = 17*V* − 229, with a correlation coefficient *R_θV_*^2^ *=* 0.9623. The applied voltage is an operational parameter that can be manipulated directly by researchers. The spreading angle is a process parameter, however it cannot be directly manipulated by certain operational parameters in relation to the working fluid’s property. Although these two different kinds of parameters have a positive correlation, the spreading angle should also receive the influence of other parameters. It should be the normal oscillation of other operational parameters (such as the shell and core fluids’ flow rates and the ambient conditions) that make the correlation coefficient vary from a number of one.

Figure 4b demonstrated the influence of applied voltage on the diameter of nanoparticles. A negative correlation is clear in that the nanoparticles’ diameter decreased as the applied voltage elevated. A linear regression suggests these two parameters have a relationship of *D* = 2713 − 82*V*, with a correlation coefficient *R_DV_*^2^ *=* 0.9624. Diameter is one of the most important parameters of the medicated nanoparticles. This equation suggests that a certain relationship exists, which can then be explored for manipulating the size of final products. This strategy has been frequently demonstrated in literature [15]. However, as seen in the relationship between spreading angle and applied voltage, the relationship between nanoparticles’ diameter and applied voltage is seemingly influenced by other parameters. Their normal oscillations would make the manipulation effect of final products’ size using the applied voltage often unsatisfactory.

Although the applied voltage did not have a strong linear relationship with the spreading angle and the nanoparticles’ diameter, the spreading angle had a better linear relationship with the size of nanoparticles, which is shown in Figure 4c. A linear equation can be regressed as *D* = 1096 − 5*θ*, with a correlation coefficient *R_D_*_θ_^2^ *=* 0.9905. The highly linear correlation between these two parameters is able to be anticipated. This is because any changes of almost all experimental parameters (including those about working fluids’ property, those about operational conditions and those about the environment) will equally exert their influences on both the atomization processes and the successive solid particles’ properties, represented by the spreading angle and the particles’ diameter.

**Figure 5:**
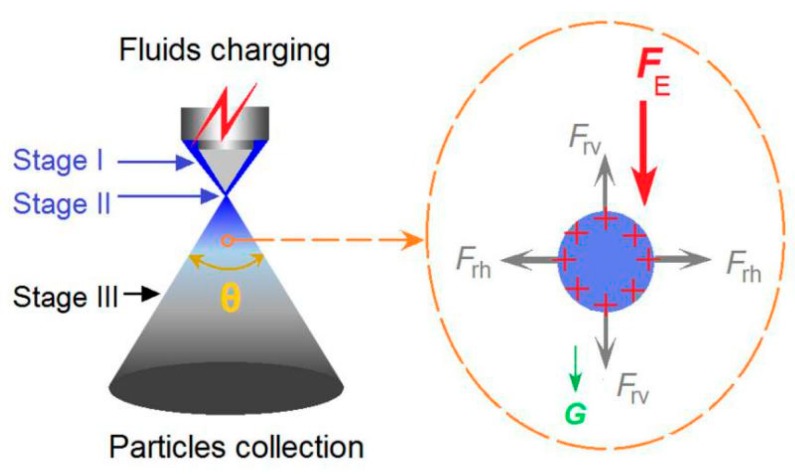
A diagram about the coaxial electrospraying and the force analysis of a charged droplet.

Despite the number of fluids an electrospraying process treats simultaneously, they will typically experience five steps, as originally demonstrated by single-fluid blending electrospraying. Shown in Figure 5, the five steps included; the fluid being charged, the formation of the Taylor cone, the convergent point of straight fluid jet, the atomization region, and the final collection of solid nanoparticles. The key intermediate steps comprise the three stages during which the working fluids are dried into solid particles. The third stage, i.e., the atomization region, often determines the solidification effects and the quality of the final solid products. Although many publications have investigated the formation of Taylor cones and the initiation of an EHDA process [49], little attention has been payed to the atomization process and which can be described by the spreading angle (*θ*).

During the atomization process, the nascent mono-disperse fluid droplets formed by the Columbic explosion should be quickly split and shrunk owing to the accumulation of surface charges and the rapid evaporation of solvents resulting from the huge surface areas. For a certain droplet, the force analysis is shown in Figure 5. The droplet should have repelling forces from all sides, for example the vertical direction repelling forces *F*_rv_ and the horizontal direction repelling forces *F*_rh_. Meanwhile, the droplet should receive the force (*F*_E_) between the two electrodes and gravity (*G*), which pushed the droplets/nanoparticles from the nozzle of the spraying head to the collector during the solidification process. When the applied voltages elevated, the droplets should have more charges, and the repelling forces should be increased correspondingly. Within a fixed collected distance, the increase of horizontal direction repelling forces *F*_rh_ should expand the atomization region and increase the spreading angle automatically. Thus, the larger applied voltage provided, the bigger the spreading angle the atomization region had. Meanwhile, the increase of applied voltage should promote the Columbic explosion and the successive splitting of droplets, resulting in nanoparticles with smaller diameters. Both the spreading angle and the nanoparticles’ diameter are the direct results of the applied voltage and are similarly influenced by the fluctuations of a series of other parameters. Thus, these two parameters have a very high correlation.

Additionally, from a standpoint of force analysis, the spreading angle is a combined effect of a series of forces under the electrical field, giving a hint that it can be a useful tool for accurately predicting the resultant nanoparticle’s diameter. This discovery is very important because new methods can now be exploited to accurately predict the size of final products and give researchers more power in respect to manipulating the electrospraying process. As far as the measurement of spreading angle is concerned, a High Frequency Camera (HFC) can be utilized to record the working process of electrospraying and should be able to improve our present work.

### 3.3. The property-Performance Relationship and the Related Mechanism

**Figure 6:**
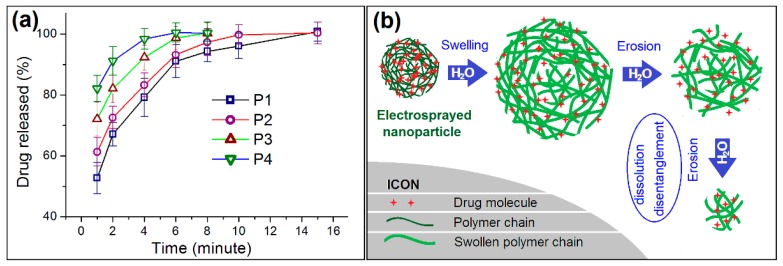
(**a**) The in vitro dissolution tests of the electrosprayed nanoparticles; (**b**) a schematic of the drug erosion mechanism from the medicated nanoparticles.

Shown in Figure 6a is the drug in vitro release profiles from the four types of medicated HPMC nanoparticles. Although all of them were able to release over 50% of the contained drug at the first minute after they were placed into the dissolution media (52.7 ± 5.1%, 61.3 ± 4.7%, 72.1 ± 5.6%, and 82.4 ± 4.3% for nanoparticles P1, P2, P3, and P4, respectively), a trend soon became clear. The finer the particles were, the faster the loaded drug was exhausted from the nanoparticles. The times needed for releasing 95% of a drug were 8.82, 6.88, 4.45 and 3.04 min for nanoparticles P1, P2, P3, and P4, respectively.

Improving the release rate and apparent solubility of poorly water-soluble drugs is always one of the major challenges in the fields of pharmaceutics and medicated nanomaterials. Traditionally, comminution of the drug powders with hydrophilic polymers such as HPMC is frequently performed to reduce the drug particle size. However, the drug fast release performance is often limited (such as IBU-HPMC xerogel granule [51]) and the preparation process is time-consuming (such as IBU-HPMC nano-suspension [52]). In comparison, the present IBU-HPMC nanoparticles were able to provide a better performance about promoting the fast dissolution of IBU and could be generated using a simple and straightforward one-step process.

HPMC is a soluble and hydrophilic polymer. During the dissolution process, the increase of diameter has both positive and negative influence on the loaded drug molecules to free into the dissolution media. Shown as Figure 6b is a diagram about the drug release mechanism. In the electrosprayed nanoparticles, the drug molecules are homogeneously distributed all over them due to the extremely fast drying process of electrospraying. When these medicated nanoparticles are placed into water, they will absorb water to swell gradually. This is a process that the water molecules penetrate in the solid nanoparticles. Meanwhile, the drug molecules should leave the polymer chains and go into the penetrated water, and further diffuse outward to the bulk solution due to the concentration gradient. This is a typical drug diffusion mechanism. As the swelling goes forward, the outer layer HMPC molecules will free into the bulk solution themselves, together with the contained and penetrated drug molecules. Thus, the erosion mechanism also happens here.

**Figure 7:**
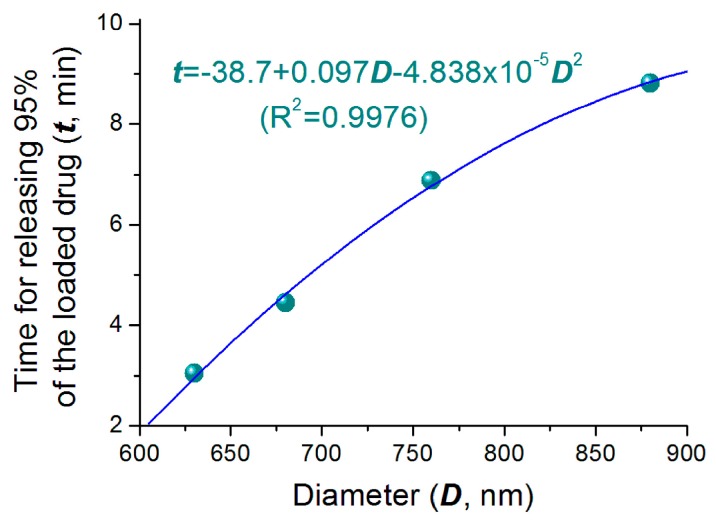
The relationship between the size of medicated HPMC nanoparticles and the time for releasing 95% of the loaded drug.

As the increase of nanoparticles’ diameters, on one hand, the penetration distance of water and diffusion distance of drug molecules should all increase correspondingly. This is to say the increase of diameter will prolong the drug release time period due to the diffusion mechanism. On the other hand, there is a competition between the swelling rate and the dissolution rate of HPMC in the gradually enlarged particles in water, the larger particles may accelerate the drug release through the easy erosion of the outer layer of the swollen particles to shorten the drug release time period. Thus, the two factors co-act on the drug release, and show a univariate quadratic equation relationship between the diameter of particle with release time apparently, as indicated in Figure 7. To fit the time needed to release 95% of the loaded drug (*t*, min) with the nanoparticles’ diameter (*D*, nm), a relationship between them can be found, i.e., *t =* 38.7 + 0.097*D* − 4.838 × 10^5^*D*^2^ (*R*^2^ = 0.9976). This univariate quadratic equation with a R^2^ value of 0.9976 suggests that the diameter of nanoparticles has a profound influence on the drug release performance. As hinted by the primary power (0.097*D*), the increase of diameter will prolong the drug release time. However, the increase of diameter may also shorten the drug release time, as suggested by the quadratic term (−4.838 × 10^5^*D*^2^). This should have a close relationship with the property of drug loaded polymer HPMC and also the density of resultant nanoparticles.

Poorly water-soluble drugs are one of the most difficult and long-existing issues in pharmaceutics [53,54,55]. Nanotechnologies have brought new lights on resolving this problem. However, how to take advantage of these advanced techniques comprises a challenge to the researchers. The present work shows a fine example to build clear process-property-performance relationship for exploring the modified coaxial electrospraying to create medicated nanoparticles. These nanoparticles can be further transferred into capsules or tablets for potential oral administration.

## 4. Conclusions and Perspectives

In the present work, the influences of spreading the angle of the atomization region (*θ*) during a modified coaxial electrospraying process on the resultant nanoparticles’ diameter, and in turn on the drug dissolution rate from the prepared IBU-loaded HPMC nanoparticles were systematically investigated. With *θ* as a key parameter, a series of process-property-performance relationships were found. These relationships include *θ* = 17*V* − 229 (*R_θV_*^2^ = 0.9623), *D* = 2713 − 82*V* (*R_DV_*^2^ = 0.9624), *D* = 1096 − 5*θ* (*R_D_*_θ_^2^ = 0.9905), and *t* = 38.7 + 0.097*D* − 4.838 × 10^5^*D*^2^ (*R*^2^ = 0.9976). Compared with the applied voltage (*V*), *θ* could provide a better correlation with the diameter of resultant nanoparticles (*D*), suggesting its usefulness for accurately predicting the nanoproducts’ size.

Today, electrospraying, is fast developing from treating a single fluid (mainly creating homogeneous particles [56,57]), to the treatment of double fluids (such as coaxial and side-by-side processes for generating core-shell and Janus particles [58,59,60]), and to the simultaneous treatment of three or even more working fluids [61,62]. In contrast, it is drawing increasing attention for producing particles on a larger-scale, just as its peer electrospinning [63,64]. Regardless of the different development directions of this advanced technique, how to build an accurate relationship between the experimental conditions and the final products’ quality always poses a big challenge to the researchers. Here, a proof-of-concept method is shown that the spreading angle, as a process parameter, can be explored to predict the resultant nanoparticles’ diameters in an accurate manner. Along this way, many new possibilities can be anticipated. For example, the precise control of the diameters of core-shell, Janus, and tri-layer nanoparticles and the size of their internal compartments. Particularly for fabrication of electrosprayed nanoparticles on a large scale, the process parameters (including those for characterizing Taylor cone and the atomization region) should be useful tools for elaborately manipulating the nanoproducts’ quality and functional performances.
